# Serum magnesium and risk of incident heart failure in older men: The British Regional Heart Study

**DOI:** 10.1007/s10654-018-0388-6

**Published:** 2018-04-17

**Authors:** Sasiwarang Goya Wannamethee, Olia Papacosta, Lucy Lennon, Peter H. Whincup

**Affiliations:** 1 Department of Primary Care and Population Health, University College London, Royal Free Campus, London, NW3 2PF UK; 2 Population Health Research Institute, St George’s University of London, Cranmer Terrace, London, SW17 ORE UK

**Keywords:** Serum magnesium, Heart failure, Coronary heart disease

## Abstract

To examine the association between serum magnesium and incident heart failure (HF) in older men and investigate potential pathways including cardiac function, inflammation and lung function. Prospective study of 3523 men aged 60–79 years with no prevalent HF or myocardial infarction followed up for a mean period of 15 years, during which 268 incident HF cases were ascertained. Serum magnesium was inversely associated with many CVD risk factors including prevalent atrial fibrillation, lung function (FEV_1_) and markers of inflammation (IL-6), endothelial dysfunction (vWF) and cardiac dysfunction [NT-proBNP and cardiac troponin T (cTnT)]. Serum magnesium was inversely related to risk of incident HF after adjustment for conventional CVD risk factors and incident MI. The adjusted hazard ratios (HRs) for HF in the 5 quintiles of magnesium groups were 1.00, 0.72 (0.50, 1.05), 0.85 (0.59, 1.26), 0.76 (0.52, 1.11) and 0.56 (0.36, 0.86) respectively [*p* (trend) = 0.04]. Further adjustment for atrial fibrillation, IL-6, vWF and FEV_1_ attenuated the association but risk remained significantly reduced in the top quintile (≥ 0.87 mmol/l) compared with the lowest quintile [HR 0.62 (0.40, 0.97)]. Adjustment for NT-proBNP and cTnT attenuated the association further [HR 0.70 (0.44, 1.10)]. The benefit of high serum magnesium on HF risk was most evident in men with ECG evidence of ischaemia [HR 0.29 (0.13, 0.68)]. The potential beneficial effect of high serum magnesium was partially explained by its favourable association with CVD risk factors. Further studies are needed to investigate whether serum magnesium supplementation in older adults may protect from the development of HF.

## Introduction

Heart failure (HF) is a major epidemic and significant public health burden in older people. After calcium, magnesium is the second most common intracellular cation and is an important electrolyte that plays a major role in metabolic processes and normal myocardial functioning, including the regulation of myocyte and endothelial cell function [1, 2]. The adverse effect of severe magnesium depletion on ventricular arrhythmias and sudden death health has long been recognised [1]. Magnesium depletion is commonly seen in HF patients [1] and is highly prevalent in the elderly due to reduced intestinal magnesium absorption and increased urinary magnesium losses [3]. In the early stages of magnesium depletion urinary magnesium concentrations are low [4]; with more marked depletion circulating magnesium concentrations (which are correlated with intracellular magnesium concentrations [2]) are affected. In recent years there has been growing evidence that lower circulating magnesium concentrations within the normal range are associated with increased risk of developing HF [2]. Experimental and clinical studies have linked hypomagnesemia to the pathogenesis of arrhythmias which is a major risk factor for HF [2] and a few population studies have shown low serum magnesium to be associated with the development of AF [5–7]. Although prospective studies and meta-analyses of prospective studies have suggested an inverse association between serum magnesium and risk of CVD [8–10], there is limited evidence on the association between serum magnesium and incident HF in the general population. However, two studies in middle-aged populations have shown low serum magnesium to be associated with increased risk of HF independent of conventional risk factors for HF and incident MI [11, 12]. The mechanisms underlying the beneficial effects of higher circulating magnesium concentrations on HF are not fully understood but are likely to be multifactorial. It has been suggested but not tested that this beneficial effect on HF is due to the beneficial effects of magnesium on the cardiovascular system, including enhancing endothelial dependent vasodilation, reducing inflammation, improving lipid and glucose metabolism and reducing cardiac arrhythmias, all factors implicated in the pathogenesis of HF [2, 13]. Whether circulating magnesium is a relevant biological marker for examining the role of magnesium in HF in older adults who are at particularly high risk of HF has not been well studied. We have therefore examined the relationship between serum magnesium and risk of incident HF in a large prospective study of older British men and explored possible mechanisms. We have examined the role of several potential pathways related to HF not previously examined including cardiac function, inflammation, endothelial dysfunction and lung function.

## Subjects and methods

The British Regional Heart Study is a prospective study involving 7735 men aged 40–59 years drawn from one general practice in each of 24 British towns, who were screened between 1978 and 1980 [14]. The population studied was socio-economically representative and comprised predominantly white Europeans (> 99%). In 1998–2000, all surviving men, then aged 60–79 years, were invited for a 20th year follow-up examination, on which the current analyses are based. Ethical approval was obtained from all relevant local research ethics committees. All men completed a mailed questionnaire providing information on their lifestyle and medical history, had a physical examination and provided a fasting blood sample. The samples were frozen and stored at − 20 °C on the day of collection and transferred in batches for storage at − 70 °C until analysis, carried out after no more than one freeze–thaw cycle. 12 lead electrocardiograms (ECG) were recorded using a Siemens Sicard 460 instrument and were analyzed at the University of Glasgow ECG core laboratory using Minnesota Coding definitions. Men were asked whether a doctor had ever told them that they had angina or MI, HF or stroke; details of their medications were recorded at the examination including use of BP lowering drugs (BNF code 3.1). 4252 men (77% of available survivors) attended for examination. Of these 4088 men provided fasting blood samples and 4031 men had blood measurement of serum magnesium.

### Cardiovascular risk factor measurements at 1998–2000

Anthropometric measurements including body weight and height were carried out. Details of measurement and classification methods for smoking status, physical activity, social class, alcohol intake, blood pressure, lung function, and blood lipids in this cohort have been described [14–17]. Serum magnesium was measured with an enzymatic colorimetric assay using a Hitachi 747 automated analyser. C-reactive protein (CRP) was assayed by ultra sensitive nephelometry (Dade Behring, Milton Keynes, UK); interleukin 6 (IL-6) was measured with ELISA (R&D Systems, Oxford, UK). von Willebrand factor (VWF) antigen was measured with enzyme-linked immunosorbent assays (DAKO, High Wycombe, UK). Predicted glomerular filtration rate (eGFR) (measure of renal function) was estimated from serum creatinine using the equation eGFR = 186 × (Creatinine/88.4)^−1.154^ × (Age)^−0.203^ [18]. Chronic kidney disease (CKD) was defined as eGFR < 60 ml/min per 1.73 m N-terminal pro-brain natriuretic peptide (NT-proBNP) was determined using the Elecsys 2010 (Roche Diagnostics, Burgess Hill, UK) [19]. Cardiac Troponin T (cTnT) was measured by a high sensitivity method on an e411 (Roche Diagnostics, Burgess Hill, UK) using the manufacturers calibrators and quality control material. The low control coefficient of variation (CV) was 6.6%, and high control CV 3.0%, and the assay limit of detection was 3 pg/ml. Evidence of myocardial ischaemia on ECG was based on Minnesota codings 1.1–1.3 (definite, probable or possible myocardial infarction) or 4.1–4.4 and 5.1–5.3 (definite, probable or possible myocardial ischaemia). Electrocardiographic left ventricular hypertrophy (LVH) was defined according to relevant Minnesota codes (codes 3.1 or 3.3). Atrial fibrillation (AF) was defined according to Minnesota codes 8.3.1 and 8.3.3.

### Follow-up

All men have been followed up from initial examination (1978–1980) for cardiovascular morbidity [14] and follow-up has been achieved for 99% of the cohort. In the present analyses, all-cause mortality and morbidity events are based on follow-up from re-screening in 1998–2000 at mean age 60–79 years to June 2014, a mean follow-up period of 15 years (range 14–16 years). Survival times were censored at date of HF, death from any cause or end of the study follow-up period (June 2014), whichever occurred first. Evidence of non-fatal MI and HF was obtained by ad hoc reports from general practitioners supplemented by biennial reviews of the patients’ practice records (including hospital and clinic correspondence) through to the end of the study period. Incident non-fatal HF was based on a confirmed doctor diagnosis of HF from primary care records and confirmed by a review of available clinical information from primary and secondary care records (including symptoms, signs, investigations, treatment response) to ensure that the diagnosis was consistent with current recommendations on HF diagnosis [20]. The incidence and determinants of HF cases identified using this process have already been reported and are consistent with results from other studies [16, 17, 19]. Incident HF included both incident non-fatal HF and death from HF (ICD 9th revision code 428 or ICD10th revision I28).

### Statistical methods

The men were divided into five approximately equally sized groups based on the quintile distribution of serum magnesium in all men without HF or MI. Cox proportional hazards model was used to assess the multivariate-adjusted hazards ratio (HR) in a comparison of the five magnesium groups using the lowest quintile as the reference group as well as in a 1 SD increase in serum magnesium. In the multivariate analysis smoking (never, long-term ex-smokers (≥ 15 years), recent ex-smokers (< 15 years), and current smokers], social class (manual vs non-manual), physical activity (inactive, occasionally active, light, moderate and at least moderately vigorous), heavy drinking (≥ 35 drinks/week), use of antihypertensive drugs (yes/no), diabetes (yes/no) and LVH (yes/no) were fitted as categorical variables. The proportional hazards assumption was examined using time-varying covariates, calculating interactions of predictor variables and a function of survival time and including them in the models. Examination of time-varying covariates indicated no violation of the proportionality assumption. Restricted cubic splines were used to visually depict the association between magnesium and incident HF. The distributions of NT-proBNP, cTnT, IL-6 and CRP were skewed and log transformation was used to normalise these factors. To evaluate whether serum magnesium predicted HF independent of incident MI during follow-up we adjusted for incident MI by fitting MI as a time dependent covariate. To calculate the excess risk explained by the mediating factors we compared the regression models with and without the mediating factors. The percent excess risk explained by the mediator is obtained by a ratio where the numerator included the difference between the unadjusted (total effect) and the adjusted (direct effect) relative risks and the denominator includes the unadjusted excess risk (total effects) [21].

### Study population

We excluded men with prior doctor diagnosis of HF or myocardial infarction (MI) (N = 508) at examination leaving 3523 men for analysis. Men with MI have very high HF incidence rates (17.40/1000/person-yrs vs 6.31/1000/per-yrs) and high prevalence of renal dysfunction which is known to influence serum magnesium levels [2]. Therefore to reduce confounding by complications of MI we excluded these men.

## Results

The mean (SD) serum magnesium in the 3523 men was 0.80 (0.07) mmol/L (range 0.48–1.06 mmol/L). 236 men (6.7%) had hypomagnesimia (< 0.70 mmol/l). During the mean follow-up period of 15 years there were 268 incident HF events (6.38/1000 person-years), 171 incident non- fatal MI (4.06/1000 person-years) and 429 incident CHD events (fatal CHD or non-fatal MI) (10.19/1000 person-years), in the 3523 men without MI or HF.

Table 1 shows baseline characteristics for each of the 5 magnesium groups. Low magnesium was significantly and strongly associated with increased BMI, heavy drinking, AF, renal dysfunction, use of antihypertensive drugs, HOMA-IR, CRP, IL-6, vWF, cTnT and NT-proBNP. Table 2 shows the association between serum magnesium and biological markers that may play a role in the association of magnesium and HF, adjusted for age and BMI in all men and excluding those with CKD. With the exception of CRP, the inverse association between serum magnesium and the CV risk factors remained after adjustment for age and BMI and upon exclusion of those with CKD.

**Table 1:** Baseline characteristics by quintiles of serum magnesium in 3523 men with no diagnosed MI or HF

	Serum magnesium (mmol/L) quintiles					*p*-trend across quintiles
	1 (< 0.75)	2 (0.75–0.79)	3 (0.80–0.82)	4 (0.83–0.86)	5 (≥ 0.87)	
No of men	610	807	688	748	670	
Age (yrs)	68.9 (5.55)	68.5 (5.60)	68.6 (5.49)	68.5 (5.37)	68.2 (5.58)	0.16
% smokers	14.6	12.9	13.0	11.8	12.1	0.14
% manual	54.9	54.5	55.4	53.6	48.8	0.03
% inactive	11.6	11.1	8.1	9.3	9.7	0.13
% heavy drinkers	4.8	4.1	4.8	3.3	2.5	0.03
% diabetes	18.7	13.4	12.1	10.0	8.7	< 0.001
% AF	3.9	3.7	4.4	2.0	2.1	0.01
% use of antihypertensive drugs	33.0	28.0	22.5	24.5	28.7	0.03
diuretics	11.2	8.4	4.9	4.9	4.8	< 0.001
% LVH	7.5	7.9	7.7	7.1	8.4	0.84
% renal dysfunction	13.7	12.9	12.9	15.1	17.5	0.01
% ECG ischaemia	24.9	24.6	22.2	20.3	19.7	0.003
BMI (kg/m^2^)	26.9 (3.87)	27.0 (3.57)	26.7 (3.56)	26.9 (3.61)	26.4 (3.24)	0.004
Heart rate (beats/min)	68.3 (14.3)	66.2 (12.0)	65.3 (12.39)	65.6 (13.22)	64.1 (11.17)	< 0.001
SBP (mmHg)	150.1 (23.8)	149.0 (23.6)	149.2 (24.0)	151.3 (23.8)	151.2 (24.4)	0.09
HDL-C (mmol/l)	1.36 (0.36)	1.33 (0.34)	1.31 (0.32)	1.33 (0.35)	1.32 (0.34)	0.02
Cholesterol (mmol/l)	5.91 (1.06)	6.00 (1.07)	6.03 (1.05)	6.16 (1.08)	6.12 (1.03)	< 0.001
HOMA	0.88 (0.81)	0.83 (0.76)	0.74 (0.71)	0.75 (0.66)	0.73 (0.71)	< 0.001
eGFR (ml/min per 1.73 m^2^)	73.3 (12.04)	72.7 (11.97)	73.52 (12.87)	72.2 (12.15)	70.83 (13.4)	0.0004
FEV_1_ (L)	2.55 (0.67)	2.59 (0.65)	2.64 (0.67)	2.66 (0.64)	2.65 (0.68)	0.001
CRP (mg/L)*	1.88 (0.82, 3.93)	1.73 (0.90, 3.49)	1.62 (0.78, 3.21)	1.55 (0.75, 3.00)	1.67 (0.80, 3.18)	0.01
IL6 (mg/L)	2.59 (1.66, 3.71)	2.48 (1.58, 3.40)	2.41 (1.57, 3.41)	2.27 (1.53, 3.14)	2.22 (1.42, 3.07)	< 0.001
vWF (IU/dL)	140.4 (48.4)	140.5 (48.4)	138.5 (44.8)	137.5 (43.9)	134.0 (43.7)	< 0.001
NT-proBNP (pg/ml)*	99.5 (44–201)	86.5 (43–172)	90.0 (47–162)	81.5 (39–148)	88.2 (41–165)	0.04
cTnT (pg/ml)*	12.55 (9.3, 16.6)	11.70 (8.4, 15.0)	11.02 (8.2, 14.6)	11.70 (8.7, 15.6)	11.47 (8.3, 15.6)	0.003
Calcium (mmol/L)	2.41 (0.10)	2.42 (0.09)	2.43 (0.09)	2.44 (0.09)	2.45 (0.10)	< 0.001
Phosphate (mmol/L)	1.13 (0.16)	1.16 (0.15)	1.15 (0.15)	1.15 (0.15)	1.18 (0.16)	< 0.001
Potassium (mmol/L)	4.36 (0.36)	4.41 (0.37)	4.45 (0.35)	4.44 (0.36)	4.47 (0.36)	< 0.001

Mean and SD; *Geometric mean and interquartile range

Heavy drinking = ≥ 35 drinks/week

*AF* atrial fibrillation, *LVH* left ventricular hypertrophy, *FEV*_*1*_ forced expiratory volume in 1 s, *CRP* c-reactive protein, *IL-6* interleukin 6, *vWF* von Willebrand factor, *NT-pro BNP* N-terminal pro-brain natriuretic peptide, *cTnT* cardiac troponin T

**Table 2:** Age-BMI adjusted Spearman’s partial correlation coefficients between magnesium and biological markers in men with no diagnosed MI or HF

	All men (N = 3523)		No CKD (N = 3505)	
	*r*	*p*	*r*	*p*
eGFR (ml/min per 1.73 m^2^)	− 0.07	< 0.0001	− 0.06	0.001
FEV_1_ (L)	0.07	0.0002	0.06	0.0008
HOMA-IR	− 0.06	0.0005	− 0.06	0.001
CRP (mg/L)	− 0.03	0.16	− 0.02	0.22
IL-6	− 0.09	< 0.0001	− 0.08	< 0.0001
vWF	− 0.05	0.004	− 0.04	0.04
Heart rate (b/min)	− 0.11	< 0.0001	− 0.10	< 0.0001
NT-proBNP (pg/ml)	− 0.04	0.02	− 0.05	0.006
cTnT	− 0.06	0.0006	− 0.07	0.0002

### Serum magnesium and incident coronary heart disease

Serum magnesium showed no association with incident CHD events. The age adjusted hazards ratio (95%CI) for the quintiles of serum magnesium were 1.00, 0.86 (0.64, 1.16), 0.86 (0.63, 1.17), 0.92 (0.68, 1.24) and 0.97 (0.71, 1.31) respectively (*p* trend = 0.99).

### Serum magnesium and incident heart failure

**Fig. 1:**
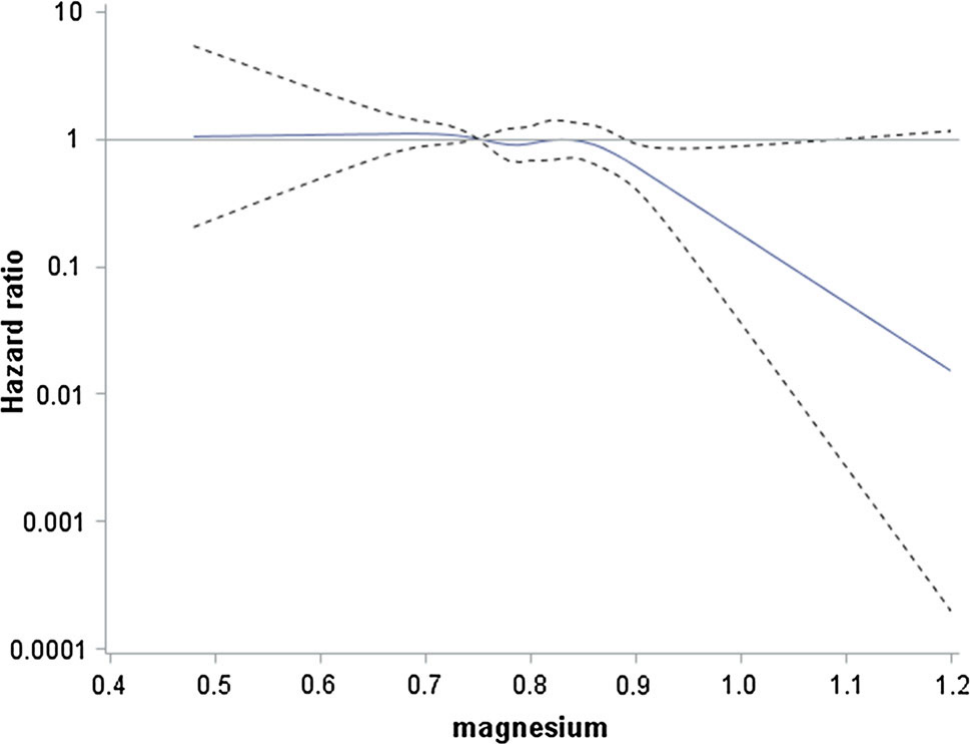
Association of serum magnesium (mmol/l) with risk of incident heart failure: magnesium modelled as restricted cubic splines with knots at the 5th (0.68 mmol/l) 20th, 40th, 60th, 80th and 95th (0.92 mmol/l) percentiles adjusted for age, smoking, physical activity, social class, BMI, diabetes, antihypertensive treatment, eGFR, LVH, heavy drinking and systolic blood pressure

In contrast to incident CHD, serum magnesium was inversely associated with risk of incident HF events after adjustment for CV risk factors; age, smoking status, social class, physical activity, heavy drinking, BMI, systolic blood pressure, HDL cholesterol, use of antihypertensive treatment, diabetes, eGFR and LVH with risk significantly reduced in those in the highest quintile (Table 3). Figure 1 shows the continuous association between serum magnesium and risk of HF after these adjustments. The Figure shows that risk declined at levels above 0.85 (~ top quintile). Exclusion of men on diuretics made little difference to the association (Table 3). Since serum magnesium showed no association with incident CHD (fatal CHD/non-fatal MI), adjustment for incident non-fatal MI made little difference to the findings (Table 3). The inverse associations with HF were attenuated after further adjustment for AF, IL-6, vWF and lung function (Table 3: models 2–4) but risk remained significantly reduced in those with elevated serum magnesium. Further adjustment for cardiac markers NT-proBNP and cTnT attenuated the association further (Table 3;models 5 and 6). However, the reduced risk seen in the top quintile was strengthened and significant when men with CKD were excluded (Table 3).

**Table 3:** Adjusted hazards ratios (95%CI) for incident HF by quintiles of serum magnesium in 3523 men with no diagnosed MI or HF

	Serum magnesium (mmol/L) quintiles						*P*-*trend*
	1 (< 0.75)	2 (0.75–0.79)	3 (0.80–0.82)	4 (0.83–0.86)	5 (≥ 0.87)	HR 1SD increase in Mg	
No of men	610	807	688	748	670		
Rate/1000 per-yrs (n)	7.9 (56)	6.7 (65)	5.8 (48)	6.7 (60)	4.9 (39)		
Age	1.00	0.75 (0.52, 1.08)	0.76 (0.51, 1.10)	0.70 (0.49, 1.01)	0.50 (0.33, 0.76)	0.84 (0.74, 0.95)	0.004
Model 1	1.00	0.74 (0.51, 1.07)	0.85 (0.57, 1.25)	0.77 (0.53, 1.14)	0.56 (0.36, 0.87)	0.88 (0.77, 0.99)	0.04
Exclude men with diuretics	1.00	0.72 (0.48, 1.08)	0.89 (0.59, 1.36)	0.79 (0.53, 1.19)	0.57 (0.36, 0.90)	0.86 (0.75, 0.98)	0.03
Model 1 +incident MI	1.00	0.72 (0.50, 1.05)	0.85 (0.59, 1.26)	0.76 (0.52, 1.11)	0.56 (0.36, 0.86)	0.87 (0.77, 0.98)	0.03
Model 2	1.00	0.72 (0.50, 1.05)	0.84 (0.57, 1.25)	0.79 (0.54, 1.16)	0.58 (0.37, 0.90)	0.89 (0.78, 1.01)	0.07
Model 3	1.00	0.74 (0.51, 1.07)	0.84 (0.57, 1.25)	0.81 (0.55, 1.19)	0.60 (0.38, 0.93)	0.90 (0.79, 1.02)	0.10
Model 4	1.00	0.75 (0.52, 1.10)	0.85 (0.58, 1.26)	0.83 (0.57, 1.23)	0.62 (0.40, 0.97)	0.91 (0.80, 1.04)	0.16
Model 5	1.00	0.74 (0.51, 1.07)	0.92 (0.62, 1.36)	0.86 (0.58, 1.27)	0.65 (0.41, 1.00)	0.93 (0.81, 1.05)	0.24
Model 6	1.00	0.79 (0.54, 1.13)	0.94 (0.63, 1.40)	0.97 (0.65, 1.43)	0.70 (0.45, 1.10)	0.95 (0.84, 1.08)	0.17
Model 6 (N = 3017;exclude men with CKD)	1.00	0.78 (0.52, 1.18)	0.78 (0.50, 1.23)	0.92 (0.59, 1.42)	0.59 (0.35, 0.98)	0.91 (0.79, 1.04)	0.17

Quintile 1 used as the reference group

Model 1 adjusted for age, smoking, social class, physical activity heavy drinking, BMI, HDL-C, diabetes, use of antihypertensive drugs, systolic blood pressure, eGFR and LVH

Model 2 adjusted for model 1 and AF

Model 3 adjusted for model 2 and IL-6 and vWF

Model 4 adjusted for model 3 and FEV_1_

Model 5 adjusted for Model 4 and cTnT

Model 6 adjusted for model 5 and NT-proBNP

We calculated the percent excess risk explained by established risk factors and potential mediators. The reduced risk explained by established risk factors (Model 1) was 12% [(0.5–0.56)/(0.5–1)*100]. Prevalent AF, lung function (FEV_1_), IL-6 and vWF together explained about 14% of the reduced risk [(0.56–0.62/(0.56–1)*100] and cardiac markers (cTnT and NT-proBNP) explained a further 21% of the reduced risk [(0.62–0.70)/0.62–1)*100]. Overall, approximately 32% of the reduced risk of HF seen for elevated magnesium appeared to be explained by AF, IL-6, vWF and cardiac markers (NT-proBNP, cTnT) combined (Model 6) after adjustment for the established risk factors [(0.56–0.70)/(0.56–1)*100].

We further examined the association between serum magnesium and HF risk separately in men with and without ECG evidence of myocardial ischemia (Table 4). The inverse association between serum magnesium and HF risk was somewhat more evident in those with evidence of myocardial ischaemia on ECG (N = 784) although a formal test for interaction was not significant (*p* = 0.21) after adjustment for factors in model 1. The association between magnesium and incident HF in those with evidence of ischaemia was not due to their higher risk of developing MI as adjustment for incident MI made little difference to the findings (Table 4).

**Table 4:** Adjusted hazards ratios (95%CI) for incident HF by quintiles of serum magnesium in men with and without evidence of myocardial ischaemia and no diagnosed MI or HF

	Serum magnesium (mmol/L) quintiles					*p*-trend
	1 (< 0.75)	2 (0.75–0.79)	3 (0.80–0.82)	4 (0.83–0.86)	5 (≥ 0.87)	
Men with no ischaemia (N = 2731)						
No of men (cases)	458 (30)	609 (43)	535 (32)	594 (39)	535 (26)	
Model 1	1.00	0.93 (0.58, 1.51)	1.02 (0.62, 1.69)	0.91 (0.55, 1.49)	0.79 (0.46, 1.36)	0.36
Model 1 +incident MI	1.00	0.93 (0.57, 1.51)	1.03 (0.62, 1.71)	0.87 (0.53, 1.44)	0.80 (0.47, 1.38)	0.38
Men with ischaemia (N = 783)						
No of men (cases)	152 (26)	197 (22)	152 (16)	151 (21)	131 (13)	
Model 1	1.00	0.49 (0.27, 0.89)	0.65 (0.35, 1.24)	0.63 (0.35, 1.24)	0.29 (0.13, 0.68)	0.03
Model 1 +incident MI	1.00	0.46 (0.25, 0.84)	0.64 (0.34, 1.22)	0.65 (0.35, 1.19)	0.28 (0.12, 0.65)	0.04

ECG not available in 11 men

Model 1 adjusted for age, smoking, social class, physical activity, heavy drinking, BMI, HDL-C, diabetes, use of antihypertensive drugs, systolic blood pressure, eGFR and LVH

### Calcium and HF

Serum calcium showed no association with incident HF. The age adjusted hazards ratio for the increasing quintiles of calcium were 1.00, 1.12 (0.77, 1.64), 1.03 (0.72, 1.48), 0.83 (0.55, 1.26) and 1.08 (0.75, 1.57) respectively.

## Discussion

In this study of older British men without history of MI or HF, serum magnesium was inversely associated with incident HF, with risk significantly reduced in those with high serum magnesium levels (upper quintile). The vast majority of these men (99%) in the top quintile of the study population had levels within the normal range (< 0.95 mmol/L) [22]. This potentially beneficial effect of high serum magnesium was more evident in those with evidence of myocardial ischaemia on ECG. No association was seen between serum magnesium concentration and incident CHD. Our findings confirm the previous 2 reports of the inverse association between serum magnesium and HF [11, 12] and extend the findings to older adults without prevalent MI. We also investigated several potential pathways including inflammation, endothelial dysfunction, cardiac function and lung function not previously assessed. Serum magnesium related inversely to prevalent AF, lung function (FEV_1_) and markers of inflammation (IL-6), endothelial dysfunction (vWF) and cardiac dysfunction (cTnT, NT-proBNP), all markers of pathways involved in the pathogenesis of HF. It was estimated that about 32% of the reduced risk of HF associated with higher serum magnesium levels within the normal range was to some extent mediated by these multiple pathways after taking into account potential confounders and established conventional CVD risk factors.

### Serum magnesium and incident CHD

Although serum magnesium was related to many risk factors for CVD, we observed no association with CHD. The lack of association between circulating magnesium concentrations and incident CHD has been reported previously [10, 23] and is consistent with the results of a meta-analysis, reporting no association between serum magnesium and CVD in men. [9] It is suggested that urinary magnesium excretion may be a more useful marker of plasma magnesium than plasma concentrations in relation to CHD [23]. Nevertheless we have observed an association between circulating serum magnesium and incident HF.

### Serum magnesium and inflammation

It has been hypothesized that the association between serum magnesium and HF may be acting through inflammation [11, 12]. Several lines of experimental evidence have suggested that higher magnesium intake may have beneficial effects on endothelial dysfunction and reducing inflammatory cytokine production [2]. We have observed an inverse relationship between circulating serum magnesium and vWF (a marker of endothelial dysfunction) and IL-6 (a pro-inflammatory cytokine and a marker of inflammation) after taking BMI into account, in line with other populations studies [24, 25]. However, we observed only weak associations with CRP a marker of low-grade chronic inflammation, which is keeping with the evidence that magnesium generally has not been found to affect markers of chronic low-grade inflammation [26]. However, the association between serum magnesium and HF was not explained by IL-6 or vWF in multivariate analysis. Magnesium levels may be impacted by renal function and diuretics. However the association was seen even after exclusion of men with diuretics and was stronger when men with CKD were excluded.

### Potential pathways relating serum magnesium concentration and HF

The association between circulating magnesium concentration and HF but not CHD suggests non-ischaemic mechanisms specific to HF are particularly important. Magnesium is known to have antiarrhythmic properties [2]. High levels of circulating magnesium may protect against the development of cardiac arrhythmias and left ventricular hypertrophy. We observed an inverse association between serum magnesium and prevalent AF, with prevalence decreased at levels above 0.83 mmol/l (2 mg/dl). These findings are consistent with the results of a recent prospective study which showed that risk of AF increased below a threshold of 1.9 mg/dl [6]. However, in the present study, the inverse association between serum magnesium and HF remained after adjustment for prevalent AF. Low magnesium has been shown to be associated with the development of AF [5–7], which in turn may lead to increased risk of HF.

Magnesium may have a direct beneficial effect on the pulmonary vasculature. Clinical studies have shown that magnesium infusion decreases pulmonary vascular resistance and pulmonary artery pressure [27, 28], factors associated with right ventricular structural abnormalities which could lead to clinical HF. In the present study, serum magnesium related inversely and strongly to lung function (FEV_1_) which is a strong predictor of HF risk [17]. Higher lung function in participants with high normal serum magnesium levels partially explained the reduced risk of HF seen in these men. Thus pulmonary function may be one of the pathways by which high levels of magnesium is beneficially related to HF.

Another possible pathway linking circulating magnesium concentrations and HF is coronary artery calcification (CAC). CAC has been shown to be associated with left ventricular diastolic dysfunction a major cause of HF with preserved ejection fraction in the elderly [29]; prospective studies have shown CAC to be associated with increased risk of incident HF [30, 31]. Animal and experimental models have shown that magnesium prevents vascular calcification in aortic vascular smooth muscle cells [32, 33]. Serum magnesium has been shown to be inversely related to CAC in the general population [34, 35]. We have shown a significant association between magnesium and cTnT which has shown to be strongly associated with the presence of CAC [36] and adjustment for cTnT attenuated the magnesium-HF association further suggesting that CAC may be another mechanism by which magnesium may protect against HF. Moreover, age has shown to be the predominant risk factor for CAC [37] and asymptomatic CHD is known to be associated with increased risk of CAC [38]. The finding that the protective effect of high serum magnesium was particularly evident in men with evidence of myocardial ischaemia suggests that magnesium may delay the progression of CAC in these high risk older men.

### Magnesium supplementation trials

The finding that low serum magnesium is associated with several adverse CVD risk factors is supported by randomised control trials. Systematic reviews of magnesium supplementation trials provide evidence as to the benefits of magnesium supplementation in reducing metabolic CVD risk factors including blood pressure and glucose as well as CRP levels [39–41]. Two recent trials have shown magnesium supplementation to improve vascular function [42, 43]. The MAGICAL-CKD trial is now underway in investigating whether magnesium supplementation can prevent the progression of CAC in subjects with chronic kidney disease [44]. However, magnesium supplementation trials on mortality outcome, largely conducted in high risk patients with acute MI have been inconclusive [13]. While one observational study has reported an inverse association between dietary magnesium intake and risk of HF hospitalisation [45], a magnesium supplementation trial has yet to be conducted in generally healthy older adults with respect to HF outcome.

### Strengths and limitations

The strengths of this study reflect its representativeness as a cohort, with a wide range of HF risk factors measured and high follow up rates. However, it was based on an older, predominantly white, male population of European origin, so that the results cannot be generalized directly to women, to younger populations or to other ethnic groups. The current findings are based on doctor diagnosed HF, which is likely to underestimate the true incidence of HF in this study population. However, the other risk factor associations to HF risk in this report and in our previous report on obesity, NT-proBNP and lung function and HF [16, 17, 19] generally accord with prior data and therefore suggest potential external validity for our findings. Information on echocardiographic measurements was not available and we were not therefore able to differentiate between systolic and diastolic HF. Serum magnesium concentration is maintained within a narrow range and values in the normal range may not fully reflect total body magnesium stores [4], although serum magnesium correlates well with intracellular magnesium levels [2]. Thus serum levels may represent the nutritional status of magnesium in the body. We had no measurements of urinary magnesium excretion, which has been suggested as a better indicator of dietary magnesium intake [23]. Serum magnesium was only measured at a single point in time, so that the strength of its association with HF may have been underestimated. Adjustments were based on measurements at examination and we had no information on incident AF which is associated with serum magnesium and HF risk. This was a prospective observation study and we cannot establish causality. Mendelian randomisation studies are needed to test for any evidence for a direct causal association between serum magnesium and HF.

### Conclusion and implications

High normal levels of serum magnesium were associated with significantly reduced risk of incident HF in older men which was partially explained by its favourable association with several potential pathways in HF including AF, inflammation, endothelial dysfunction, lung function and cardiac dysfunction. Serum magnesium may be modifiable by magnesium intake raising the possibility that magnesium may be a modifiable risk factor for HF. Intervention trials are needed to confirm whether magnesium supplementation in older adults, in particular those with evidence of myocardial ischaemia, would reduce the risk of HF.
